# Immunomodulation and T Helper TH_1_/TH_2_ Response Polarization by CeO_2_ and TiO_2_ Nanoparticles

**DOI:** 10.1371/journal.pone.0062816

**Published:** 2013-05-08

**Authors:** Brian C. Schanen, Soumen Das, Christopher M. Reilly, William L. Warren, William T. Self, Sudipta Seal, Donald R. Drake

**Affiliations:** 1 Sanofi Pasteur, VaxDesign Campus, Orlando, Florida, United States of America; 2 Department of Molecular Biology and Microbiology, Burnett School of Biomedical Science, UCF College of Medicine, Orlando, Florida, United States of America; 3 Advanced Materials Processing and Analysis Centre (AMPAC), Department of Mechanical, Materials and Aerospace Engineering (MMAE), Nanoscience and Technology Center (NSTC), Orlando, Florida, United States of America; 4 Virginia College of Osteopathic Medicine Research, Virginia-Maryland Regional College of Veterinary Medicine Virginia Tech, Blacksburg, Virginia, United States of America; Johns Hopkins School of Medicine, United States of America

## Abstract

Immunomodulation by nanoparticles, especially as related to the biochemical properties of these unique materials, has scarcely been explored. In an *in vitro* model of human immunity, we demonstrate two catalytic nanoparticles, TiO_2_ (oxidant) and CeO_2_ (antioxidant), have nearly opposite effects on human dendritic cells and T helper (T_H_) cells. For example, whereas TiO_2_ nanoparticles potentiated DC maturation that led towards T_H_1-biased responses, treatment with antioxidant CeO_2_ nanoparticles induced APCs to secrete the anti-inflammatory cytokine, IL-10, and induce a T_H_2-dominated T cell profile. In subsequent studies, we demonstrate these results are likely explained by the disparate capacities of the nanoparticles to modulate ROS, since TiO_2_, but not CeO_2_ NPs, induced inflammatory responses through an ROS/inflammasome/IL-1β pathway. This novel capacity of metallic NPs to regulate innate and adaptive immunity in profoundly different directions via their ability to modulate dendritic cell function has strong implications for human health since unintentional exposure to these materials is common in modern societies.

## Introduction

Nanoparticles (NPs) have become a ubiquitous staple of modern life, yet researchers have a less than complete understanding of how these materials affect human health. In fact, it is becoming increasingly clear that NP species with distinct physiochemical properties (size, shape, composition, solubility, surface chemistry, etc.) can interact with body systems in a variety of different ways. For instance, CeO_2_ NPs have shown great promise at protecting tissues from oxidative stress and have been proposed as a modality to alleviate healthy tissue damage during cancer radiation therapy [1]–[3]. On the other hand, metallic NPs have been shown to negatively impact human health by inducing acute toxicity in the lung and kidneys [4]. As well, metallic NPs have been found to induce the production of pro-inflammatory cytokine when delivered *in vivo*, which suggests these materials likely can engage cells of the immune system.

Despite multiple studies detailing the influence of size, solubility, and surface modification on the biocompatibility of metallic nanoparticles [5], far fewer reports have directly examined how the varied physical characteristics of these NPs affect their interaction with the human immune system. Published work from our laboratory and other groups has suggested the inflammatory potential of metallic NPs is inversely proportional to their sizes [6]–[8]. Other studies have shown the highly charged surface of some metallic NPs can facilitate their binding to proteins and other molecules, leading to macromolecular complex formation and/or altered protein conformations that can be highly immunogenic [9]. Some authors have also suggested redox-active surface groups can directly influence NP interactions with immune cells, but the impact of these studies is dampened because catalytic NPs were not directly compared against NPs with opposite redox activities [10]–[14]. Indeed, few studies to date have examined whether antioxidant NPs affect immune function.

Considering the various NP physiochemical properties that could be considered impactful on immune function, redox activity is perhaps the most important since catalytic NPs have a unique capacity to directly modulate reactive oxygen species (ROS). (ROS are well-established regulators of immune reactions [15].) To formally address whether catalytic activity affects NP-immune interactions, we performed a comprehensive examination of the immunomodulatory potential of two metallic NPs (TiO_2_ and CeO_2_) with opposing redox activities in an *in vitro* model of human immunity. This system, which encompasses a number of modular constructs that permit the evaluation of different facets of immunity, has been shown in a variety of published studies to support the generation of responses that reflect known human *in vivo* immune profiles against a series of biologic compounds and vaccines [8], [16]–[26].

Specifically for this study, we have examined the effect of these unique NP species on human immune cell viability, phenotype, uptake, ROS production, and function in the *in vitro* cell culture model. Intriguingly, we noted that the reductive CeO_2_ NPs were uniquely capable of stimulating DCs to produce IL-10, and when co-cultured with T cells, triggered a strong T_H_2-biased/regulatory cytokine profile. In contrast, oxidative TiO_2_ NPs induced DCs to produce IL-12 and polarized T cells toward a T_H_1-biased program. As a whole, these data provide evidence that NPs have the potential to modulate human DC and T helper cell function with a directionality that is linked to surface redox properties and suggest a novel basis for modulating immunity via NPs with tunable surface chemistries.

## Results

### NP Characteristics

**Figure 1:**
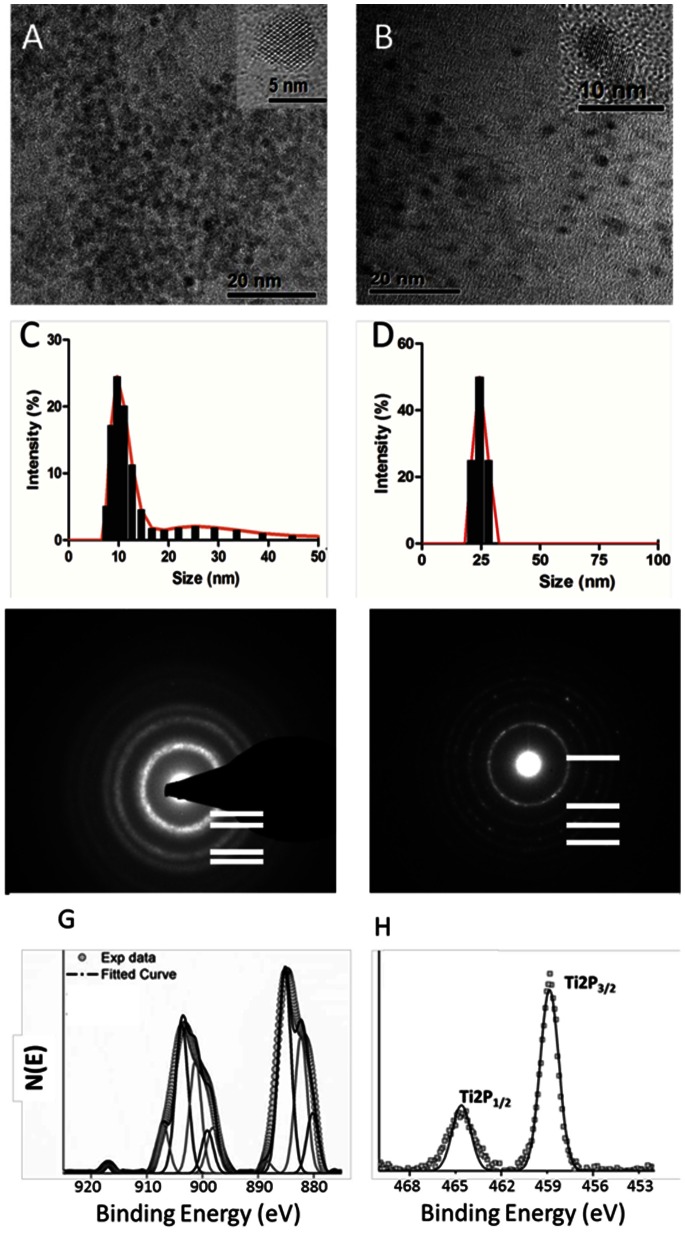
CeO_2_ NPs and TiO_2_ NPs appear as soft agglomerates when diluted in X-VIVO 15 serum free media. High resolution transmission electron microscopy of (A) CeO_2_ NPs indicates a composition of individual 3–5 nm nanocrystallites and (B) 7–10 nm TiO_2_(anatase) NPs. The average size distribution of (C) CeO_2_ and (D) TiO_2_ NPs were measured using dynamic light scattering following a 24 hour incubation of the prepared NP solutions (each at 500 µM) in X-VIVO 15. Selected area electron diffraction patterns (SAEDP) of the CeO_2_ (E) and TiO_2_ NPs (F) were carried out using a high-resolution transmission electron microscope (HRTEM) equipped with a FEI Tecnai F30 having an energy-dispersive X-ray (EDX) analyzer. The SAED pattern of CeO_2_ NPs, where A(111), B(200), C(220) and D(311) correspond to the different lattice planes of CeO_2_ and confirms the crystalline structure of this material. Similarly, the SAED pattern of TiO_2_ also confirms the crystalline nature of the material since the A(101), B(004), C(200) and D(211) rings correspond to the different lattice planes of the NPs. Surface oxidation state of CeO**_2_** and TiO**_2_** NPs were calculated from the XPS spectrum of Ce3d (G) and Ti 2p (H). (G) Deconvoluted peaks at 882.36 eV, 898.20 eV, 901.23 eV, 907.03 eV, and 916.64 eV are attributed to a Ce^4+^ oxidation state (light gray solid line) while 880.22 eV, 885.24 eV, 899.16 eV and 903.68 eV are the characteristic peaks of a Ce^3+^ oxidation state (dark gray solid line). Intensity of the peaks for Ce3+ and Ce4+ were estimated, and Ce^3+^/Ce^4+^ ratio on the surface of the nanoparticles were calculated and found to be 1.66. (H) In the case of TiO_2_ NPs, the binding energies of Ti 2p_3/2_ and Ti 2p_1/2_ are at approximately 458.84eV and 464.62 eV, respectively. The difference of ∼5.8 eV in both peaks indicates a valence state of +4 for Ti on the surface of the NPs.

To determine whether surface catalytic activity can affect the interaction of NPs with the immune system, we performed a parallel evaluation of the capacity of TiO_2_ and CeO_2_ NPs, which have opposite catalytic activities, to stimulate immune cell activation in an *in vitro* model of the human immune system. The physical properties of the CeO_2_ and TiO_2_ NPs included in this study are discussed in detail in the *Materials and Methods* section and are summarized in Table 1. Since we were specifically interested in understanding whether catalytic activity impacts the interaction of metallic NPs with the immune system, we first needed to ensure other physical features of these NPs, such as agglomeration and purity, did not contribute to changes in immune function in the *in vitro* model. As shown in Figure 1, the CeO_2_ and TiO_2_ NPs had a tendency to form soft agglomerates of 10 and 25 nm in diameter, respectively, when cultured for 24-hr in X-VIVO-15 tissue culture media (serum-free culture media used in all of the biological assays discussed below). Additionally, we confirmed both NP preparations were free of contaminating LPS (EU <0.05) that could otherwise compromise the outcome of the subsequent immunoassays (see Figure S1).

**Table 1:** Physical properties of nanomaterials included in this study.

Particles	Preparation Method	Diameter nm)	DLS Peak intensity	BET Surface (m^2^/g)	Zeta Potential (mV)*	Surface Reactivity	Crystal Structure
TiO_2_	HT-WCS^1^	7–10†	25 nm	239	−9.84±0.19	Oxidative	Anatase
CeO_2_	RT-WCS^2^	3–5†	10 nm	90	−10.01±1.50	Reductive	Fluorite

^1^High temperature wet chemical synthesis.

^2^Room temperature wet chemical synthesis.

* Zeta potential after 24 hrs in X-VIVO 15 culture media.

† Average diameter of NPs, expressed as mean size ± SD nm.

### DC Cytotoxicity and Maturation Resulting from NP Treatment

**Figure 2:**
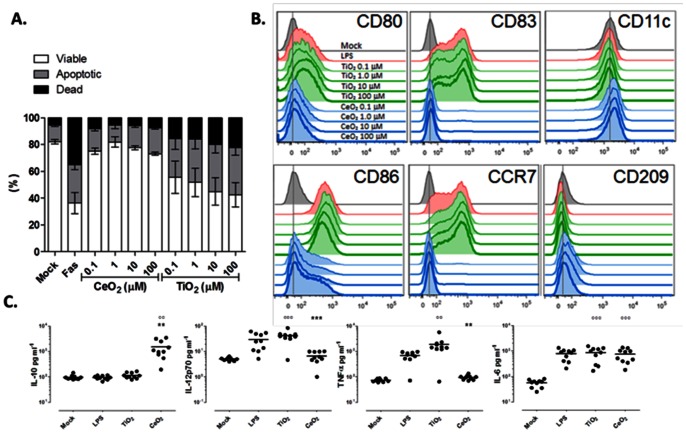
CeO_2_ NPs trigger human DCs to produce significant amounts of IL-10. (A) Dendritic cells were exposed to the indicated concentrations of NPs for 24 hrs and assessed for viability using 7-AAD and apoptosis by Po-Pro staining. As negative and positive controls, DCs were left untouched (mock) or treated with 1 µg/ml Fas ligand (FAS), respectively. Bar graph data are plotted as mean ±SD. (B) Dendritic cells were exposed to the indicated concentrations of NPs for 24 hours and assessed for phenotypic expression of human DC markers, as indicated, by flow cytometric analysis. (C) Supernatants from DCs stimulated with 1 µM of either NPs were examined for soluble cytokines by Bio-Plex assay. Each dot on the scatter plot represents the signal for an individual donor; Data are mean+/−SD, n = 10. A paired t-test was performed: **p<0.005, ***p<0.0005 versus TiO_2_ or CeO_2_ group; °°p<0.005, °°°p<0.0005 versus mock group.

We recognize NPs can potentially interact with a variety of immune cell populations, but focused our initial evaluation on DCs since they are involved in many facets of innate and adaptive immunity. We previously established a dose range for TiO_2_ NPs in our *in vitro* immune cell model [8]; here, we started here by establish whether assay-derived APCs had a similar tolerance forCeO_2_ NPs. Following a 24-hr treatment of the DCs with NPs, the cells were labeled with a fluorescent apoptotic dye (PO-PRO), in combination with a vital dye (7-AAD), to discriminate between live, dead, and apoptotic cells. Unlike TiO_2_ NPs, which triggered appreciable apoptosis and death of the cultured DCs in a dose-dependent manner, we observed no increase in apoptosis or death in DCs cultured with CeO_2_ NPs (Figure 2A). It is important to note that several published articles have shown these NPs do not interfere with these standard fluorescent readouts [3], [8], [27], [28]. To further mitigate the risk of NP interference with these assays, the nanoparticles were diluted in, or delivered, in cultures maintained in the presence of protein containing media and the cells were thoroughly washed in protein containing buffers prior to their acquisition by any instrument. While our findings on TiO_2_ NP cytotoxicity in human DCs are consistent with our previous work and the observations of others using cell lines [8], [29]–[31], we are unaware of other studies demonstrating a high tolerance of human DCs for CeO_2_ NPs.

Metallic NPs have previously been shown to activate/mature DCs towards an enhanced functional state [8], [32]. To determine whether this DC immunostimulatory potential was driven, at least in part, by the oxidative activity of TiO_2_ NPs, we directly compared DC activation/maturation triggered by TiO_2_ and the antioxidant CeO_2_ particles. As shown in Figure 2B, DCs treated with as little as 1 µM TiO_2_ NPs increased their expression of surface receptors involved in T cell priming/activation (HLA-DR, CD80 and CD86) and migration (CCR7). TiO_2_-treated DCs also upregulated surface CD83, a phenotypic hallmark of DC maturation, but only at the highest treatment dose (100 µM). Interestingly, a 24-hour exposure of the DCs to even the highest dose of CeO_2_ NPs had almost no effect on CCR7, CD83, CD80, CD86, or HLA-DR expression levels.

Besides triggering changes in surface marker expression, maturation stimuli also often cause DCs to produce a variety of soluble and membrane-bound cytokines that modulate many facets of innate and adaptive immunity. Indeed, TiO_2_ particles stimulated a strong cytokine response from the DCs that was of a pro-inflammatory slant (Figure 2C, IL-12, TNFα) and consistent with the phenotype changes highlighted in Figure 2B. Considering the lack of DC surface marker changes triggered by CeO_2_ (Figure 2B), we were surprised to find these NPs induced the APCs to produce significant quantities of the immunoregulatory cytokine, IL-10. However, the inability of CeO_2_ NPs to activate DCs may not be surprising in light of the observation that antioxidants, such as *N*-acetylcysteine, do not induce DC maturation, and to some extent, have even been shown to mitigate DC maturation [33], [34]. Furthermore, some published studies have also shown chemical antioxidants, like phenyl *N-tert*-butyl nitrone, have the propensity to induce IL-10 production in cultured DCs [35], [36].

### Redox Potential as a Regulator of DC Activation State

**Figure 3:**
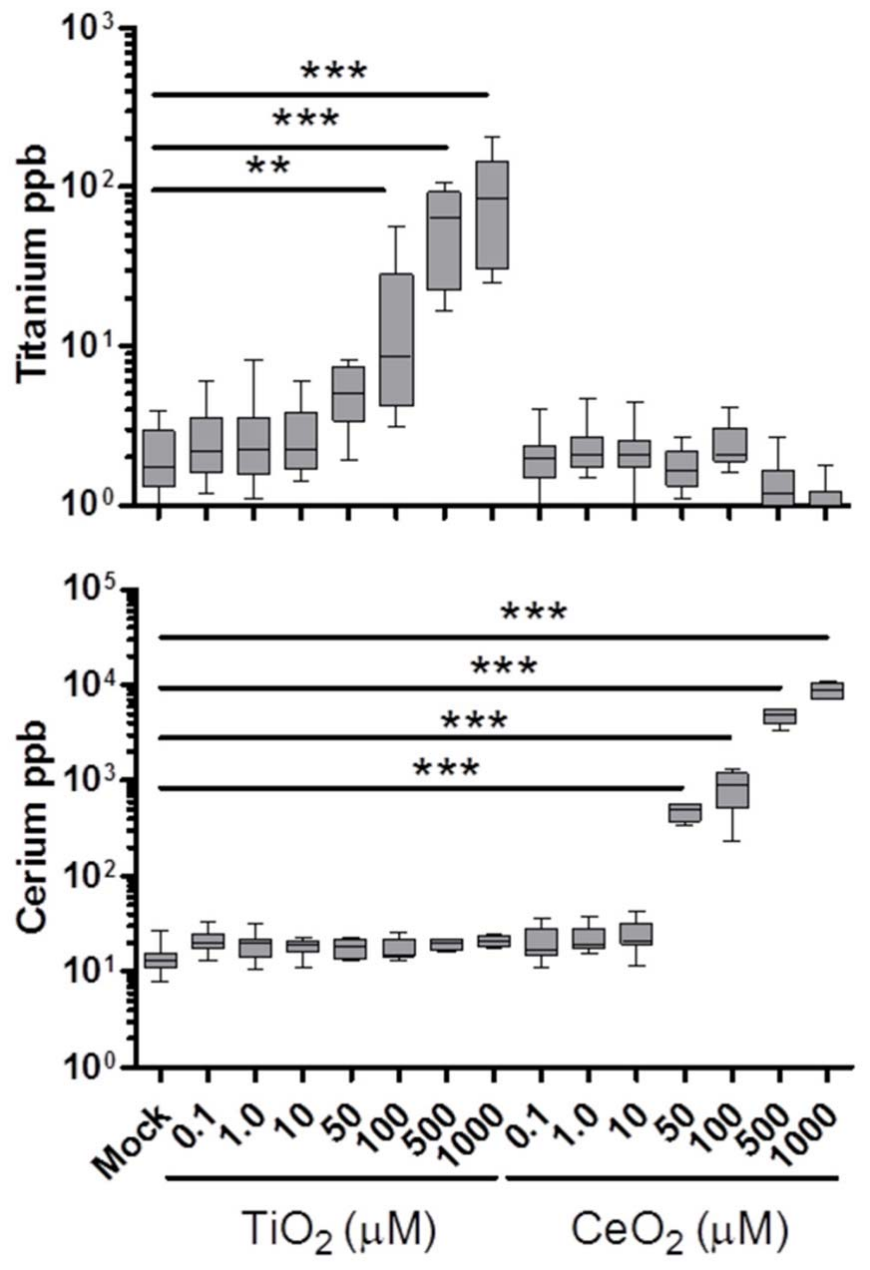
Human DCs have the capacity to internalize CeO_2_ and TiO_2_ NPs. Cytokine-derived human DCs were pulsed for 24 hours with the listed dosing range of either NP. The DCs were harvested and washed several times before examination by inductively coupled plasma-mass spectroscopy (ICP-MS) for metal analysis and detection (ppb). Each sample was examined for the presence of both cerium (bottom) and titanium (top) as an assay detection control. Ten donors were analyzed in total. The paired t-test was used for statistical analyses. n = 10; **p<0.005, ***p<0.0005 versus mock group.

Considering evidence suggesting oxidative stress can result in cytotoxicity and inflammation [37], we suspected the differential responses generated by TiO_2_ and CeO_2_ NPs might be explained by their opposite surface reactivity. To rule out the possibility that these distinct responses could be explained simply by the differential uptake of TiO_2_ and CeO_2_ NPs by DCs, we used a highly sensitive inductively coupled plasma-mass spectroscopy (ICP-MS) technique [38], [39] to examine whether the NPs were localized within the treated DCs. With this technique, we were able to rule out uptake as an explanation for the results of Figure 2 since Figure 3 shows uptake is dose-dependent and detectable by ICP-MS at concentrations above 50 µM for both NPs species. While previous studies examined APC-mediated uptake of TiO_2_ and CeO_2_ at a much higher dose ranges than those used in the current study [40]–[43], it should be noted that we used a lower treatment dose range because we wanted to ensure that the immune cells remained viable for subsequent functional assessments.

**Figure 4:**
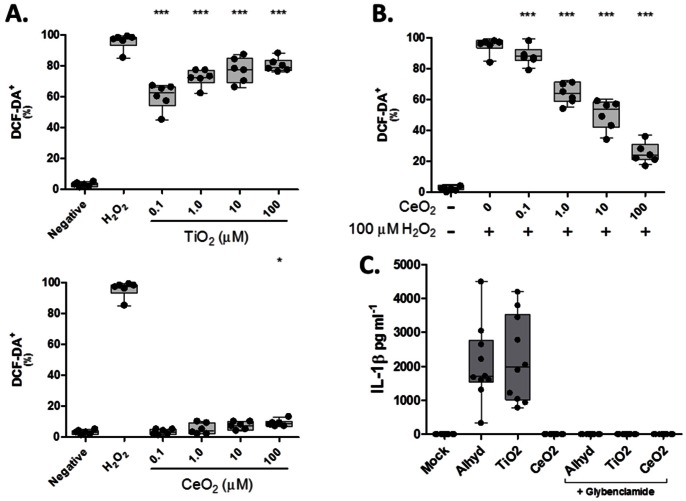
Redox activities of nanomaterials modulate ROS production and NLRP3 inflammasome activation in DCs. (A) Human DCs were cultured in the absence or presence of the indicated doses of TiO_2_ or CeO_2_ NPs for 24 hr prior to being examined for their production of ROS. (B) DCs were cultured in the presence of cerium oxide at various concentrations for 8 hours and then H_2_O_2_, an inducer of ROS, was added for the remainder of the 24 hour incubation period. Oxidative stress was measured by DCF-DA staining of ROS. Six donors where examined in total. (C) DCs were stimulated for 24 hours with Alhydrogel (AlHy, 150 µg/ml) as a positive control for NLRP3 activation. Alternatively, TiO_2_ NPs or CeO_2_ NPs were delivered at 1 µM to the cultures for 24 hours prior to being measured for the presence of IL-1β in the presence or absence of NLRP3 inhibitor, glybenclamide (50 µM). Each data point is representative of an individual donor, n = 10. A paired t-test was performed: **p<0.005, ***p<0.0005 versus mock group.

As noted in the *Introduction* section, catalytic NPs have a unique capacity to directly modulate reactive oxygen species (ROS). Given our findings thus far, and the known redox activity these materials possess, we felt it necessary to examine ROS as a possible mechanism to explain the unique and disparate DC activation/maturation profiles triggered by TiO_2_ and CeO_2_ NPs. Towards this goal, we analyzed intracellular oxidative stress levels in NP-treated DCs with a specific dye, DCF-DA, which fluoresces upon contact with ROS. Figure 4A reveals that TiO_2_ NPs induced human DCs to generate ROS in a dose-dependent manner and at levels comparable to the positive control, H_2_O_2_. In contrast, CeO_2_ NPs triggered little or no ROS in treated DCs and were even capable of blunting ROS production in DCs treated with H_2_O_2_ (Figures 4A and 4B). (It should be noted that H_2_O_2_-induced ROS production was unaffected by TiO_2_ treatment.).

Although ROS can act through a variety of downstream pathways to regulate/potentiate immune reactions, perhaps its most important feature is its ability to activate innate danger sensors, such as the NLRP3 inflammasome [44]. Since the detection of IL-1β has been routinely used as a readout of NLRP3 inflammasome activation [44], we used this cytokine as an indirect measure of whether TiO_2_ and/or CeO_2_ NPs activate the NLRP3 inflammasome in human DCs. Based on past studies demonstrating TiO_2_ NPs activate the NLRP3 inflammasome in mice [44], we were not surprised to find DCs treated with these NPs were stimulated to secrete heightened quantities of IL-1β. In subsequent studies, we showed the selective NLRP3 inhibitor, glybenclamide (50 µM), abolished IL-1β production in these cultures. This provides further evidence that TiO_2_ NPs act through the NLRP3 inflammasome to induce IL-1β production (Figure 4C). In stark contrast to these results, we found CeO_2_ NPs triggered no IL-1β production by the cultured DCs (Figure 4C), which further supports our earlier conclusions that these anti-oxidant NPs induce a null or anti-inflammatory response in DCs.

### NPs Drive CD4^+^ T Cell Proliferation and T_H_1/T_H_2 Polarization

**Figure 5:**
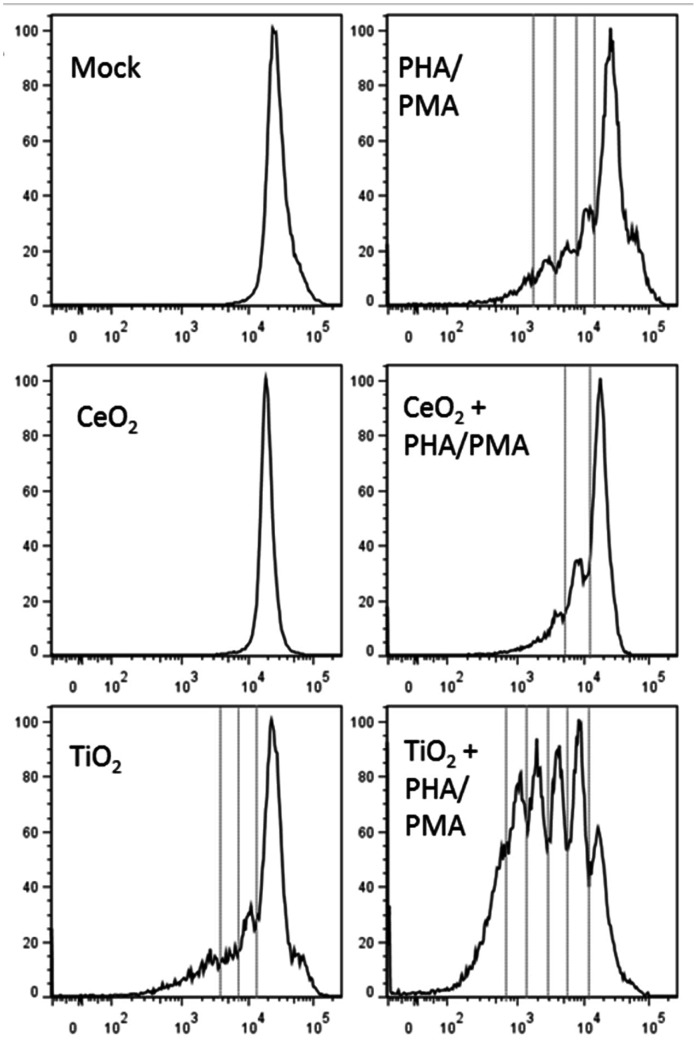
TiO_2_ and CeO_2_ NPs induce differential T cell responses. CD4^+^ T cells were labeled with the division-sensitive dye, CFSE, and cultured in the presence or absence of the indicated stimuli (NPs: 10 µM, PHA: 1 µg/mL, PMA: 50 ng/mL) for 5 days. Thereafter, the cells were harvested and examined for proliferating (CFSE-low) cells by flow cytometry. Histograms are representative plots from one of the five donors investigated, CFSE plotted on x-axis as a percent of maximum (y-axis).

Following our finding that CeO_2_ and TiO_2_ provide human DCs with distinct stimulatory/maturation cues, we questioned whether these differences would, in turn, translate into unique patterns of T cell responses resulting from stimulation with the NP-treated DCs. Prior to addressing this issue, we first investigated whether NPs directly activate lymphocytes in a 5-day stimulation assay where T cell proliferation serves as the primary readout of the response. To our surprise, TiO_2_ had a modest immunostimulatory effect on the T cells, as demonstrated by their capacity to induce an increase in the divided (CFSE-low) lymphocyte population over the untreated control. Furthermore, the co-administration of TiO_2_ NPs with the mitogens, PHA and PMA, synergistically increased the proliferative response (Figure 5). CeO_2_ NPs alone did not induce measurable T cell proliferation but, interestingly, did reduce the proliferative response when added with the mitogen cocktail (Figure 5). Of note, neither of the particle types affected the viability of the T cells over a broad dose range (see Figure S2). As an additional measure to investigate the stimulatory effect these NPs have on T cells, we examined the expression levels of CD95 (FasR), which becomes upregulated under stress or disease conditions and is part of the programmed death response [45]. The expression of CD95 was unaffected by either NP treatment (see Figure S3). However, treatment with TiO_2_ NP in addition to the mitogen cocktail, PHA/PMA, revealed the capacity for TiO_2_ NPs to drive a much stronger level of CD95 expression as compared to CeO_2_ NP and mitogen treated T_H_ cells (Figure S3). While this evidence doesn’t tell us precisely how these NPs are interacting with T cells, the NPs are affecting T cell phenotype and function as measured by these assays.

To better define the impact of catalytic NPs on human adaptive immunity, we directly examined the capacity of NP-treated DCs to stimulate naïve T cell responses using our *in vitro* model of human immunity. With this approach, the engagement of TCR by foreign HLA class II molecules on the surface of mismatched DCs is sufficient to induce the activation of the lymphocytes in an antigen-independent fashion. Here, DCs were left untouched (iDC), matured with a maturation cocktail (mDC, positive control), or primed with CeO_2_ or TiO_2_ NPs before being co-cultured with allogeneic CD4^+^ T cells. After 5 days, the cells and culture supernatants were harvested for evaluation by flow cytometry (cell viability and proliferation) and Bio-Plex assay (cytokine production).

**Figure 6:**
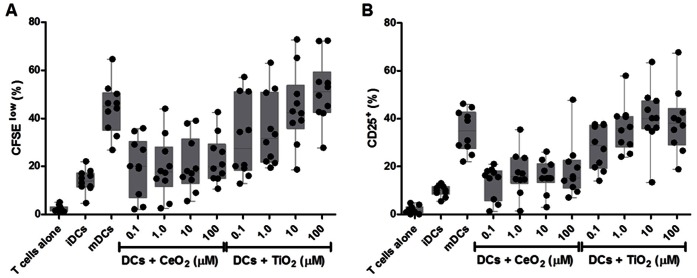
CeO_2_ and TiO_2_ NP-primed DCs differentially modulate CD4^+^ T cells proliferation. Naïve CD4^+^ T cells were isolated and labeled with the division-sensitive dye, CFSE. (A) The CFSE-labeled T cells were then co-cultured for 5 days with immature DCs (iDCs; untreated), matured DCs (mDCs; treated overnight with TNFα and PGE_2_), or NP treated DCs (24 hour treatment with the indicated nanomaterial described on the x-axis). (B) Thereafter, the cells were harvested and examined for proliferating (CFSE-low; left panel) and activated (CD4^+^CD25^+^; right panel) T cells by flow cytometry, n = 10. See Table S1 and Table S2 for Tukey’s honest significance test for pairwise comparisons of each treatment.

**Figure 7:**
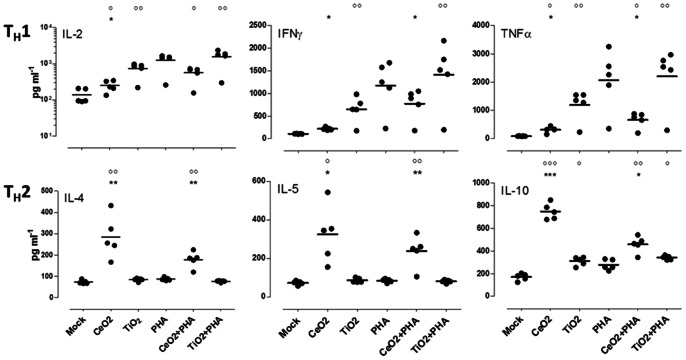
CeO_2_ and TiO_2_ NPs induce naïve human CD4^+^ T cells towards distinct cytokine profiles. DCs were treated with NPs (10 µM) for 24 hours prior to being harvested, washed and co-cultured with a mismatched (allogeneic) donor purified T cells over a 5-day incubation period. T cells were cultured with PHA (1 µg/mL), where indicated. Supernatants from the T cell stimulation assays were examined for T_H_1 and T_H_2 associated cytokines by Bio-Plex array. Each dot on the scatter plot represents the signal for an individual donor. Five donors were examined in total. A paired t-test was performed: *p<0.05, **p<0.005, ***p<0.0005 versus TiO_2_ or CeO_2_ group; °p<0.05, °°p<0.005, °°°p<0.0005 versus mock group.

Although the CeO_2_ NP-treated DCs had little influence on allogeneic naïve CD4^+^ T cell proliferation, TiO_2_ NP-treated DCs boosted the magnitude of the proliferative response (Figure 6). As well, we observed that both particles triggered cytokine responses, but the profiles were nearly opposite: TiO_2_ NPs-pulsed DCs triggered a pro-inflammatory T_H_1-biased cytokine response (IL-2, IFN-γ) while DCs pulsed with CeO_2_ NPs induced a naïve T cell response dominated by T_H_2 cytokines (IL-4, IL-5, and IL-10) that are predominately anti-inflammatory and promote humoral-skewed responses. Beyond their capacity to participate in the induction of a T_H_2-biased T cell response, the CeO_2_ NPs were even capable of eliciting the production of IL-4, IL-5 and IL-10 in T cell co-cultures stimulated with a strongly Th1-biasing mitogen (Figure 7). While we might have anticipated that a well-described pro-inflammatory particle like TiO_2_ could drive a type 1 immune response, the response profile induced by CeO_2_ NPs, including IL-10 secretion by DCs (Figure 2) and T_H_2 polarization (Figure 6 and 7),suggest a unique functional property of metallic antioxidant NPs that, to our knowledge, has not previously been described.

## Discussion

Despite the emergence and rapid adoption of NPs into modern life, a paucity of data exists on how these materials influence human physiology, including the immune system. In an earlier publication from our laboratory, we showed particle size had a profound impact on the ability of TiO_2_ NPs to induce inflammation in an *in vitro* model of human immunity [8]. In the current study, we questioned whether other physiochemical features of NPs, specifically surface reactivity, might also influence the immunomodulatory potential of NPs. Towards this goal, we employed the same *in vitro* model employed above to examine whether oxidative TiO_2_ and anti-oxidative/reductive CeO_2_ NPs have altered capacities to influence human immune reactions.

In a series of experiments aimed at examining the impact of these NPs on innate responses, we demonstrated that TiO_2_ NPs push human DCs towards a more activated/pro-inflammatory state while CeO_2_ NPs triggered a more anti-inflammatory profile in these cells. Given these observations, we were not surprised to see the NP-treated APCs, in turn, triggered nearly opposite T helper cell response profiles (CeO_2_ promoted a T_H_2 profile while TiO_2_ lead to a T_H_1 pattern). Our current results with TiO_2_ NPs were consistent with our published work and reports by others showing these NPs can induce oxidative stress and inflammation [8], [46], [47]. On the contrary, we did not anticipate CeO_2_ NPs would induce such a pronounced T_H_2-biased (IL-4, IL-5 and IL-10) T cell response and even blunt mitogen-induced T_H_1 (IL-2, IFNγ, and TNFα) cytokine production. Though the overall profile of cytokines produced by T_H_ cells stimulated with CeO_2_-stimulated DCs is consistent with a T_H_2 profile, it should also be noted that the strong IL-10 response might be reflective of T_reg_ induction in these cultures. Additionally, we examined additional cytokines/chemokines which were not activated in response to treatment (See Methods section). Our preliminary results did not indicate CeO_2_-stimulated cultures yield a higher frequency of T_reg_ cells, but further experimentation will be necessary to fully investigate this possibility.

To date, few studies have detailed the capacity of NPs to polarize T_H_ cell response and none have shown the pronounced NP-induced T_H_ biasing demonstrated here. For example, Liu et al. showed poly-hydroxylated metallofullerenol NPs could induce T_H_ cytokine responses, but only in a mixed fashion (both T_H_1 and T_H_2 cytokines were produced). In a second example, PLGA-NPs were shown to push T_H_ cells towards a specific cytokine profile, but only in cases where the NPs were conjugated to known T_H_ biasing peptides [48], [49]. This unique and pronounced T_H_ response polarization resulting from metal-oxide (TiO_2_ and CeO_2_) NP treatment could possibly be explained by the differences in the capacities of the two NP species to regulate ROS production, particularly since ROS can function as a second messenger and modulator of immunity [15], [50]–[52]. Going a step further, it is interesting to speculate the anti-oxidant redox activity of CeO2 NPs triggers significant IL-10 production by the DCs that ultimately leads to the strong IL-10 response by activated T cells in the DC/T cell cultures since this cytokine is a well-established regulator of T_H_ cell differentiation [53]–[55]. This hypothesis is consistent with prior studies showing ROS-generating materials, like TiO_2_ NPs, trigger downstream pro-inflammatory effects and antioxidants prevent the initiation of the innate immunity in LPS-stimulated macrophages, as evidenced by the suppression of pro-inflammatory cytokine (TNF-α, IL-1β) secretion by the treated cells [56], [57]. As well, it is supported by another study showing palladium NPs, a reducing agent with anti-oxidative properties similar to CeO_2_ NPs, can trigger IL-10 production by human peripheral blood mononuclear cell (PBMCs) [58]. It should be noted that our observations suggesting NPs modulate immune function through an ROS pathway does not preclude the possibility that the particles act on APCs via other mechanisms.

While no current *in vitro* culture model can replicate all the intricacies and variables of the *in vivo* environment, we have assessed these materials to the best of our capacities using our *in vitro* model which has provided meaningful pre-clinical information on human immune responses shown to be reflective of human responses in prior publications [59], [60]. However, since these materials are unable to be tested in a clinical setting, we are in the process of validating our *in vitro* results through the use of a murine model. Unfortunately, such evaluations are very complex and require a great deal of consideration across a number of experimental parameters including dosing schema, diluent, route of administration, number of treatments, kinetics, (disease) model, and possible readouts.

We speculate the distinct immunostimulatory potentials observed between CeO_2_ and TiO_2_ are likely explained by the distinct manner in which these materials are able to absorb photons. Here, the materials differ in that the photons have a tendency to migrate to the surface of TiO_2_ NPs, where they are free to react with oxygen, water, or hydroxyls to form free radicals [61]. On the other hand, the CeO_2_ NPs absorb these free photons where they remain isolated from the outside environment [61]. In fact, this chemistry leads to their distinct oxidant/antioxidant properties, as illustrated in Figure 4A and 4B, where ROS production by DCs increased linearly with TiO_2_ NP dose, but remains absent in CeO_2_ NPs-treated cultures. Moreover, CeO_2_ actually inhibited ROS production induced by H_2_O_2_ in a dose-dependent manner, which suggests this NP species is a very potent anti-oxidant.

Taken as a whole, the results of this study suggest differences in surface reactivity can profoundly affect how metallic NPs interact with the human immune system (Table 2). Specifically, these data suggest low-dose exposure of human immune cells to redox-active NPs have the propensity to modulate human innate and adaptive immunity, i.e, DC activation and primary CD4 T helper cell differentiation state. For this reason, CeO_2_ NPs (and perhaps other anti-oxidant moieties) might offer researchers a unique opportunity to push adaptive responses in a focused direction away from a T_H_1 bias and towards a T_H_2/T_reg_ bias. Alternatively, TiO_2_ might serve as a potent Th1-promoting treatment during prophylaxis or disease treatment. On the contrary, the immunomodulatory potential of NPs could pose a considerable health risk if encountered in an uncontrolled environment. Specifically, the T_H_-skewing potential of NPs could possibly translate into effects on general inflammatory diseases, airway hyperresponsiveness, asthma, and autoimmunity. With further study, features like catalytic behavior may potentially be exploited for engineered NPs to meet a particular goal, such as enhancing immune responses during vaccination or mediating immune tolerance against allergies or autoimmune disease.

**Table 2:** Immunological and biochemical effect of nanomaterials investigated.

Particles	Surface Reactivity	Cytokines induced	Inflammasome induction	T cell proliferation	TH polarization	ROS
TiO2	Oxidizing	Proinflammatory	Yes	Modest	TH1	Generator
CeO2	Reducing	Anti-inflammatory	No	None	TH2	Scavenger

## Materials and Methods

### Subjects

This study included PBMC blood product from 10 healthy donors. This study was approved by the ethics committee of the Chesapeake Research IRB. Full documentation of application process, orientation attendance, and signed written informed consent forms were obtained from all donors prior to their participation and the study procedures were conducted in accordance with the Declaration of Helsinki (protocol CRRI 0906009). All applicants have met guidelines set forth in the approved IRB protocol, which includes (but is not limited to) restrictions regarding general health, disease screening, weight, and age. Blood collections were performed at Florida’s Blood Centers (Orlando, FL), a state/federally regulated blood collection center, using standard techniques approved by their institutional review board. The PBMCs collected under our donor program are collected, stored, and later used for various immunological research projects at Sanofi Pasteur VaxDesign Campus. The donors’ PBMCs used in this study were randomly selected from our cryo-bank.

### Reagents

Bacterial lipopolysaccharide (LPS), phytohaemagglutinin (PHA), and phorbol 12-myristate 13-acetate (PMA) were obtained from Sigma (St. Louis, MO). ROS levels were determined using the fluorescent label, 2-,7-dichlorodihydrofluorescein diacetate (DCF; Sigma). Glybenclamide was purchased from Sigma and used as an NLRP3 inflammasome inhibitor [62].

### Synthesis of NPs

TiO_2_ NPs were synthesized by wet chemical synthesis as previously described [8]. Briefly, a 50∶50 mixture of ultrapure ethanol (Sigma) and deionized water (18.2 M) was boiled to reflux. The pH of the boiling solution was adjusted to 3.0 with the addition of 1 N HCl. Titanium isopropoxide (Sigma) was added slowly to the refluxing mixture, which precipitates immediately to a white solution. The solution was then stirred at 85°C for 4 hours. The white solution was then cooled to room temperature and washed several times with ethanol until dry. The final preparation was mostly anatase (partially amorphous) TiO_2_. CeO_2_ NPs were synthesized using wet-chemical synthesis as described previously [63]. Briefly, cerium nitrate hexahydrate was dissolved in deionized water (18.2 MΩ). A stoichiometric amount of hydrogen peroxide was added as an oxidizer and immediately resulted in the formation of cerium oxide NPs. The NP powder was obtained by washing the precipitate of CeO_2_ NPs several times with acetone and water to remove the surfactant used in the synthesis process. The solution was aged further to allow the slow reduction of surface cerium from 4^+^ oxidation state to 3^+^ oxidation state in acidic medium by maintaining the pH of the suspension below 3.5 with nitric acid. Nanoparticle treatments investigated in this study are reported in molarity and the mass per volume is indicated in parenthesis as follows: TiO_2_ - 0.1 µM (0.0079 µg/mL), 1.0 µM (0.0798 µg/mL), 10 µM (0.798 µg/mL), 50 µM (3.993 µg/mL), 100 µM (7.986 µg/mL), 500 µM (39.93 µg/mL), 1000 µM (79.86 µg/mL); CeO_2_ - 0.1 µM (0.0172 µg/mL), 1.0 µM (0.172 µg/mL), 10 µM (1.72 µg/mL), 50 µM (8.605 µg/mL), 100 µM (17.2 µg/mL), 500 µM (86.05 µg/mL), 1000 µM (172.11 µg/mL).

### Characterization

TiO_2_ and CeO_2_ NPs were analyzed using high-resolution transmission electron microscopy (HRTEM; Philips 300 TECNAI operated at 300 kV) to confirm their shape, size, and morphology. The HRTEM samples were prepared by dipping a polycarbon-coated copper grid into a dilute suspension of NPs dispersed in acetone. The surface area of the NPs were measured based on physical adsorption of ultra-high purity nitrogen gas at liquid nitrogen temperature using a Brunauer-Emmett-Teller (BET) Nova 4200e instrument manufactured by Quantachrome (Boynton Beach, FL). The samples were prepared in quartz tubes and degassed at 240°C in vacuum for 3 hours before actual measurement. The size of the NPs was determined by the dynamic light scattering method using the Zetasizer Nano manufactured by Malvern Instruments (Worcestershire, UK). The physical characterization of the materials is reviewed in Figure 1 and summarized in Table 1.

### Evaluation of Endotoxin Contamination

All NP preparations were confirmed negative for the presence of endotoxin contamination using the FDA-approved Endosafe LAL colorimetric and turbidimetric assay system (Charles River Laboratories, Wilmington, MA). This data is shown in Figure S1.

### PBMC Isolations

Within hours following their harvest from the donor, the enriched leukocytes were centrifuged over a Ficoll-plaque PLUS (GE Healthcare, Piscataway, NJ) density gradient [24], [25]. PBMCs at the interface were collected, washed, and cryopreserved in IMDM media (Lonza, Walkersville, MD) containing autologous serum and DMSO (Sigma-Aldrich, St. Louis, MO).

### Generation of Cytokine-Derived DCs

DCs used throughout the assays of this study were prepared using our previously published methodology [25]. Briefly, monocytes were purified from total PBMCs by positive magnetic bead selection (Miltenyi Biotec, Cologne, Germany) and cultured for 7 days in X-VIVO 15 (Lonza) serum-free media supplemented with GM-CSF (R&D Systems, Minneapolis, MN) and IL-4 (R & D Systems). In all assay conditions described below, treatments were delivered on day 6 followed by harvesting on day 7 for incorporation into the various assays.

### ROS Determination

DCs were treated with serial dilutions of TiO_2_ NPs and CeO_2_ NPs for 24 h. Subsequently, the cultures were washed and treated at room temperature for 30 min with DCF at a final concentration of 10 µM. The cells were washed of excess dye with DPBS, harvested using cell-dissociation solution (Sigma), and washed again in DPBS. Fluorescence in the FITC channel from absorbed and oxidized DCF (indicative of peroxide levels) was analyzed by flow cytometry using an LSR II (Becton Dickinson). FlowJo software (Treestar, Ashland, OR) was used for data analysis.

### DC Phenotype/cytokine Analysis

For flow cytometry analysis of surface molecule expression, DCs were washed in fluorescence-activated cell sorting buffer (FACS Buffer). Fc receptors were blocked with mouse serum (Jackson ImmunoResearch, West Grove, PA) to prevent nonspecific binding. DCs were then stained with a vital dye (LIVE/DEAD®; Invitrogen). (Conversely, for determination of apoptosis DCs were stained with Po-Pro/7-AAD (Invitrogen) at the end of the surface antibody staining.) After washing away excess viability dye with PBS, the cells were then incubated with the appropriate antibody cocktail. The antibodies used in the staining panels include HLA- DR, CD14, CD40, CD80, CD83, CD86, CD19, CD3, CD209, and CCR7. All antibodies we purchased from eBioscience (San Diego, CA) with the exception of CD209 (BD Pharmigen, San Diego, CA). Following staining, cells were washed in FACS buffer and immediately acquired on a BD LSRII flow cytometer (Becton Dickinson), and data analyzed using FlowJo software V9.2 (Tree Star).

Supernatant from the treated DC culture wells and DC:T cell co-cultures were collected and analyzed for cytokine production by means of the Bio-Plex Multiplexing array system (Bio-Rad, Hercules, CA) as previously described [8]. The Bio-Plex array used in this study included: IL-1ra, IL-1β, IL-2, IL-4, IL-5, IL-6, IL-7, IL-9, IL-10, IL-13, IL-15, IL-17, IFN-gamma, eotaxin, G-CSF, GM-CSF, MIP-1α, MIP-1β, PDGF-BB, RANTES, TNF-alpha, and VEGF.

### NP Uptake by DCs

Samples treated for 24-hrs with TiO_2_ or CeO_2_ NPs were harvested, washed and placed in 70% nitric acid overnight and then microwaved to digest the cellular material. The temperature of the cell harvest was steadily increased to 200°C over a 20-mins period and held constant at 200°C for an additional 20 minutes. The samples were then boiled down to less than 1 ml and reconstituted in water to an exact volume of 10 ml. Titanium and cerium levels were assessed using inductively coupled plasma mass spectroscopy (ICP-MS) using published techniques that have been optimized to minimize the possibility of surface-bound or aggregated NPs from being carried over from the washing steps [39].

### CD4^+^ T Cell Proliferation Assay

Human CD4^+^ T cells were isolated from PBMCs by positive selection using EasySEP CD4^+^ T cell isolation kit II (Stem Cell Technologies, Vancouver, Canada). The purified CD4^+^ T cells were then carboxyfluorescein succinimidyl ester (CFSE)-labeled to follow proliferation and incubated either in the presence of the described NPs with or without PHA/PMA or without stimulation and left in culture for 5 days. The cells were harvested, and examined by flow cytometry using LIVE/DEAD AQUA and CFSE (Invitrogen) and antibodies specific for CD4 and CD25 (eBioscience) by flow cytometry.

### In vitro Model of Human Immunity

Dendritic cells were generated using a 3-dimensional tissue engineered construct described previously [8], [24]. These DCs were either untouched, matured with a cocktail of TNFα and PGE_2_ as described previously as a positive control [24], or were exposed to various doses of NPs for 24 hours prior to being harvested. The treated DCs were harvested and added at an optimized ratio of 1∶400 to allogeneic naïve CD4^+^ T cells isolated using EasySEP CD4^+^ T cell isolation kit II (Stem Cell Technologies) and labeled with CFSE (Invitrogen). After five days the cultures were harvested and stained for CD25, CD3, CD4, (eBioscience) and Live/Dead Aqua for viability (Invitrogen) and then acquired by flow cytometry using BD Pharmingen’s LSR II as described above. Additionally, supernatant’s were collected and examined for cytokine secretion by Bio-Plex array as previously described above. Here, PHA/PMA (1 µg/mL; 50 ng/mL) was used not only as a positive control for cytokine production, but also added in combination with NP-treated DC co-cultures where described.

### Flow Cytometry, Data Plotting and Statistical Analysis

Cytometry data was analyzed using FlowJo software V9.2 (Tree Star). Each experiment was repeated with at least three donors or more, as described in the figure legends and plotted as an average (with S.D.) or displaying each data point. Analyzed statistical results were determined using a paired Student’s t-test. Statistical significance was considered at p<0.05 or otherwise stated in figure legend. Tukey’s honest significance test was employed, in conjunction with an ANOVA, to determine if the treatment groups (between CeO_2_ and TiO_2_) are significantly different from each other. All graphs and biostatistics were produced using GraphPad Prism software V5 (La Jolla, CA).

## Supporting Information

Figure S1 **Endotoxin levels of CeO_2_ and TiO_2_ NP measured <0.05 EU/mL.** The TiO_2_ and CeO_2_ NPs were diluted to 100 µM concentrations in sterile endotoxin-free water. The diluted preparations were then examined for endotoxin levels using an automated FDA-licensed endotoxin detection system by Charles Rivers Laboratories. No detectable (ND) levels of endotoxin were observed in the NP preparations. Three independent samples were run to generate average bar with S.D.
(TIF)

Figure S2 **T cells remain viable following treatment with NPs.** Freshly isolated CD4^+^ T cells were cultured in the absence or presence of TiO_2_ NPs (1 µM), CeO_2_ NPs (1 µM), PHA/PMA (as a positive assay control), or combinations of either NP with PHA/PMA. After 5 days, the cultures were harvested and stained with the viability dye (LDA) and examined by flow cytometry. The % LDA negative represents the fraction of live cells in the culture. Each column is the average of 5 donors plotted with S.D.
(TIF)

Figure S3 **CeO_2_ mediates cellular stress induced by mitogen control as indicated by reduced CD95 expression.** Freshly isolated CD4^+^ T cells were cultured in the absence or presence of TiO_2_ NPs (1 µM), CeO_2_ NPs (1 µM), PHA/PMA (as a positive assay control), or combinations of either NP with PHA/PMA. After 5 days, the cultures were harvested and stained with anti-CD95 and assessed by flow cytometry. The mean fluorescent intensity of the CD95 expression was calculated in FlowJo and plotted. Each column is the average of 5 donors plotted with S.D. (p<0.05 where noted).
(TIF)

Table S1 **Statistical analysis of** **Figure 6** **A.** Tukey’s honest significance test was employed, in conjunction with an ANOVA, to determine if the treatment groups (between CeO_2_ and TiO_2_) are significantly different from each other in relation to CFSE fluorescence.
(DOCX)

Table S2 **Statistical analysis of** **Figure 6** **B.** As in Table S1, Tukey’s honest significance test was employed, in conjunction with an ANOVA, to determine if the treatment groups (between CeO_2_ and TiO_2_) are significantly different from each other in relation to CD25 expression.
(DOCX)
