# Supplementary figures and images for: Immunomodulation and T Helper TH1/TH2 Response Polarization by CeO2 and TiO2 Nanoparticles
Source: PLoS One. 2013 May 8;8(5):e62816. doi: 10.1371/journal.pone.0062816 (PMC3648566; doi:10.1371/journal.pone.0062816)

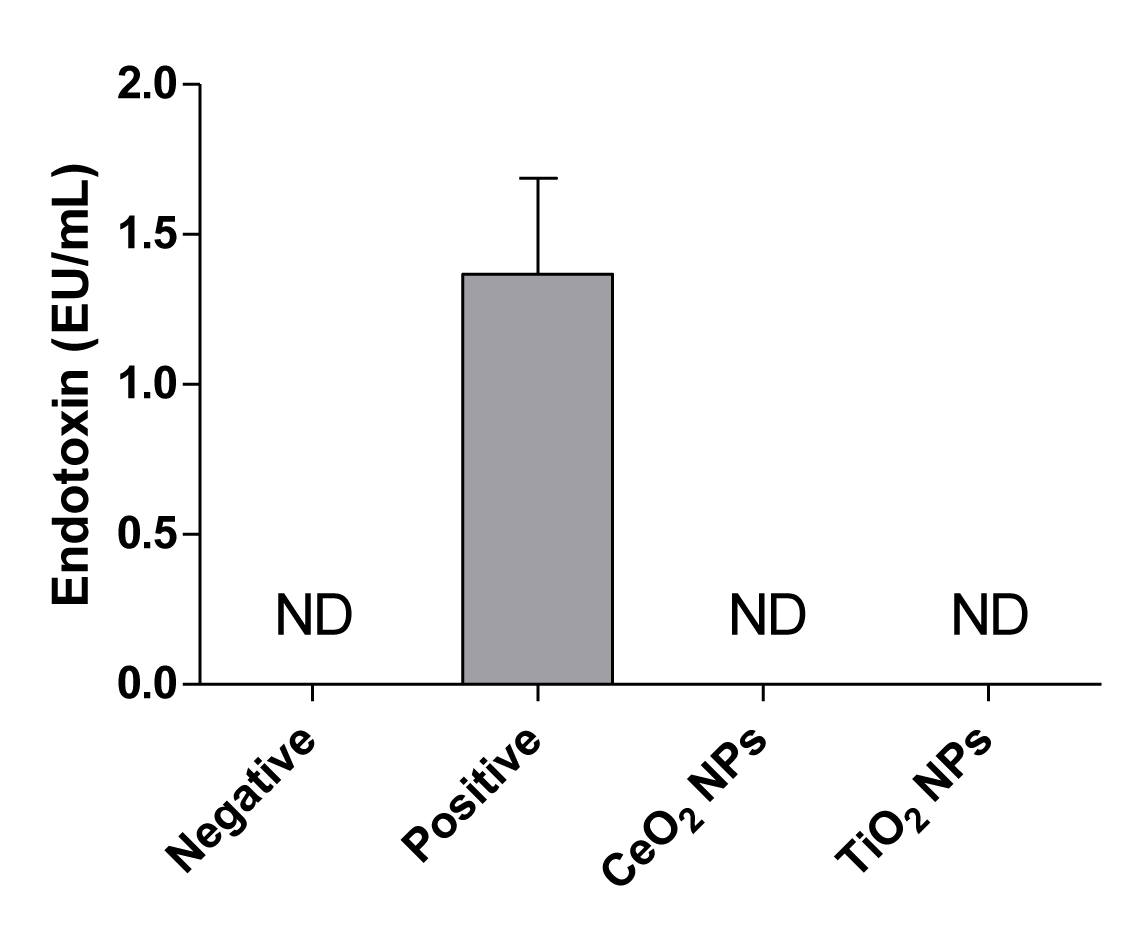

Supplement: Figure S1 — Endotoxin levels of CeO2 and TiO2 NP measured <0.05 EU/mL. The TiO2 and CeO2 NPs were diluted to 100 µM concentrations in sterile endotoxin-free water. The diluted preparations were then examined for endotoxin levels using an automated FDA-licensed endotoxin detection system by Charles Rivers Laboratories. No detectable (ND) levels of endotoxin were observed in the NP preparations. Three independent samples were run to generate average bar with S.D. (TIF) [file pone.0062816.s001.tif]

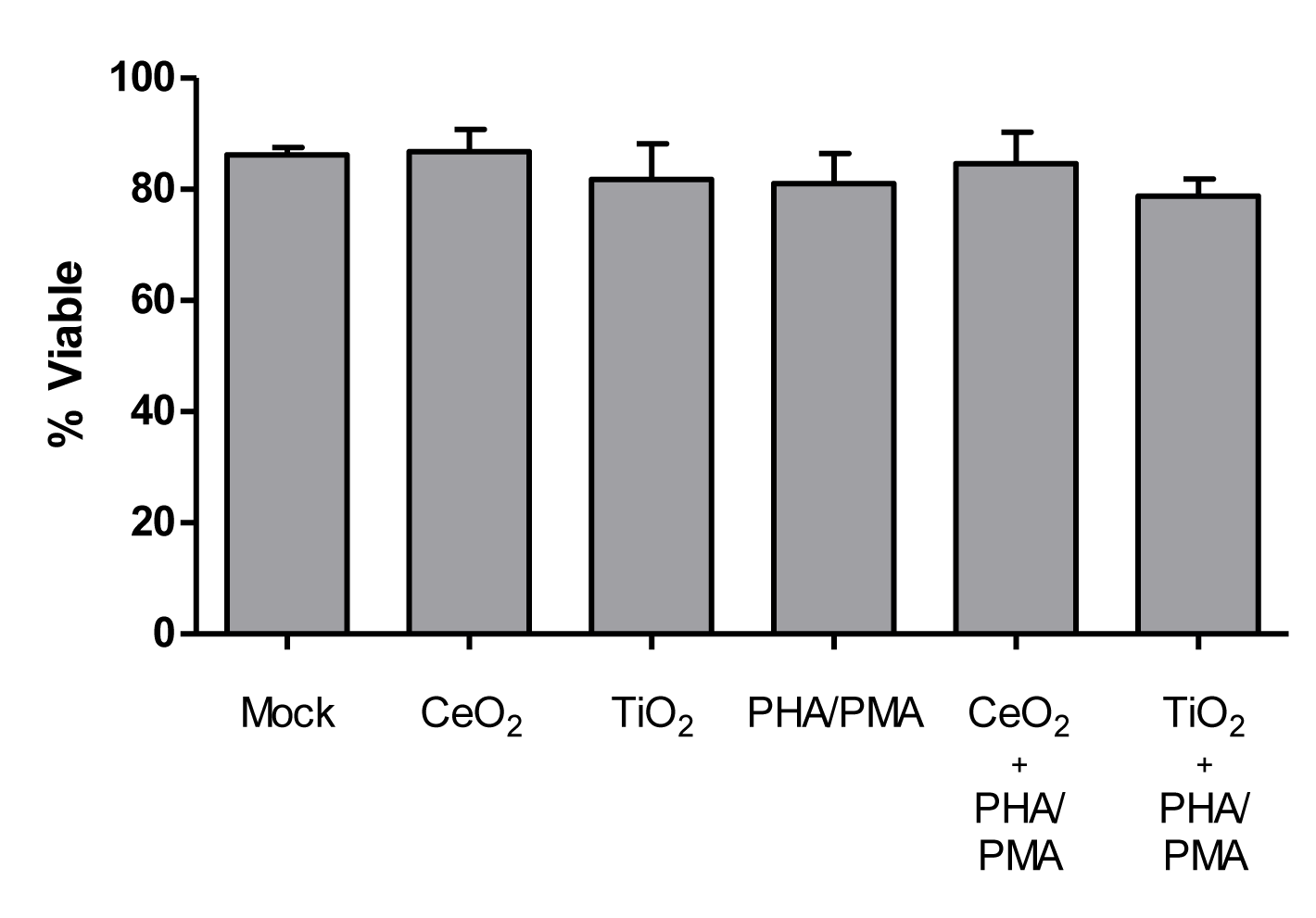

Supplement: Figure S2 — T cells remain viable following treatment with NPs. Freshly isolated CD4+ T cells were cultured in the absence or presence of TiO2 NPs (1 µM), CeO2 NPs (1 µM), PHA/PMA (as a positive assay control), or combinations of either NP with PHA/PMA. After 5 days, the cultures were harvested and stained with the viability dye (LDA) and examined by flow cytometry. The % LDA negative represents the fraction of live cells in the culture. Each column is the average of 5 donors plotted with S.D. (TIF) [file pone.0062816.s002.tif]

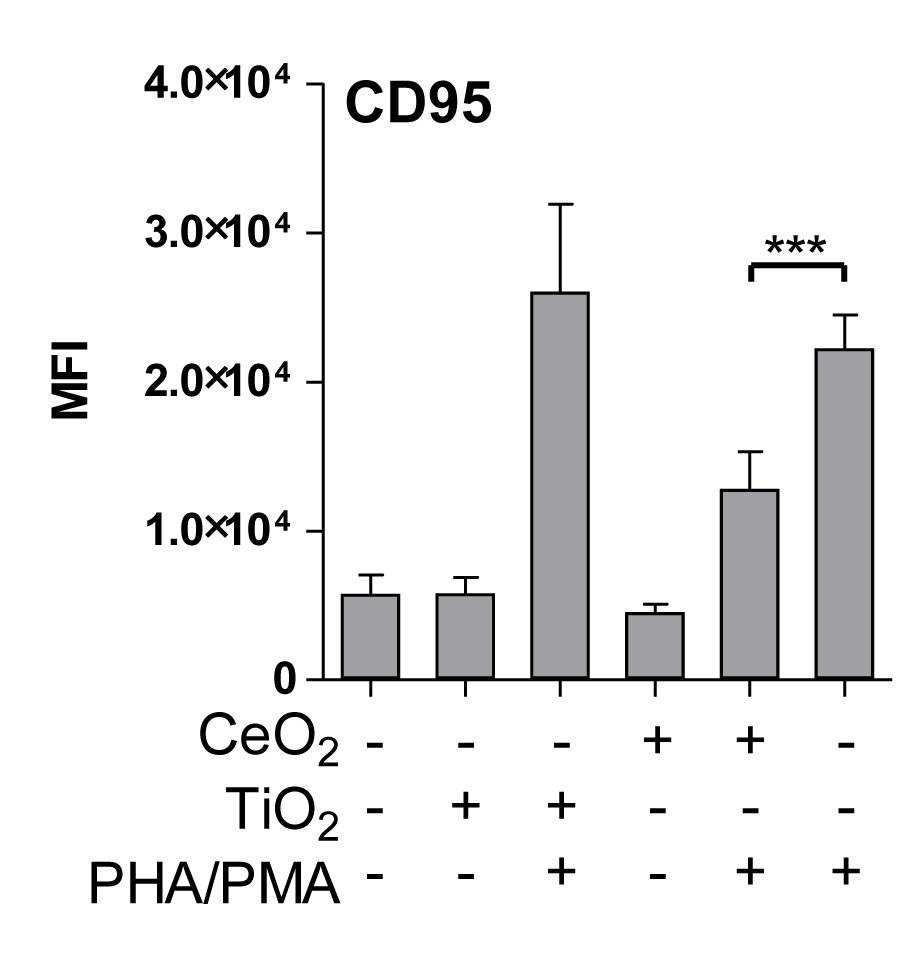

Supplement: Figure S3 — CeO2 mediates cellular stress induced by mitogen control as indicated by reduced CD95 expression. Freshly isolated CD4+ T cells were cultured in the absence or presence of TiO2 NPs (1 µM), CeO2 NPs (1 µM), PHA/PMA (as a positive assay control), or combinations of either NP with PHA/PMA. After 5 days, the cultures were harvested and stained with anti-CD95 and assessed by flow cytometry. The mean fluorescent intensity of the CD95 expression was calculated in FlowJo and plotted. Each column is the average of 5 donors plotted with S.D. (p<0.05 where noted). (TIF) [file pone.0062816.s003.tif]
