# Supplementary material for: Immunomodulation and T Helper TH1/TH2 Response Polarization by CeO2 and TiO2 Nanoparticles
Source: PLoS One. 2013 May 8;8(5):e62816. doi: 10.1371/journal.pone.0062816 (PMC3648566; doi:10.1371/journal.pone.0062816)
Supplement: Table S1 — Statistical analysis of Figure 6 A. Tukey’s honest significance test was employed, in conjunction with an ANOVA, to determine if the treatment groups (between CeO2 and TiO2) are significantly different from each other in relation to CFSE fluorescence. (DOCX) [file pone.0062816.s004.docx]

Supplementary Table S1. Statistical analysis of Figure 6 A.

| **Tukey's Multiple Comparison Test** | **Mean Diff.** | **q** | **Significant? P < 0.05?** | **Summary** | **95% CI of diff** |
| --- | --- | --- | --- | --- | --- |
| **T cells alone vs iDCs** | **-11.69** | **4.25** | **No** | **ns** | **-24.59 to 1.202** |
| **T cells alone vs mDCs** | **-41.82** | **15.2** | **Yes** | ******* | **-54.71 to -28.92** |
| **iDCs vs mDCs** | **-30.12** | **10.95** | **Yes** | ******* | **-43.02 to -17.23** |
| **iDCs vs 0.1 µM CeO_2_** | **-5.868** | **2.133** | **No** | **ns** | **-18.76 to 7.026** |
| **iDCs vs 1.0 µM CeO_2_** | **-5.468** | **1.987** | **No** | **ns** | **-18.36 to 7.426** |
| **iDCs vs 10 µM CeO_2_** | **-6.448** | **2.344** | **No** | **ns** | **-19.34 to 6.446** |
| **iDCs vs 100 µM CeO_2_** | **-8.808** | **3.202** | **No** | **ns** | **-21.70 to 4.086** |
| **iDCs vs 0.1 µM TiO_2_** | **-17.69** | **6.431** | **Yes** | ******* | **-30.59 to -4.800** |
| **iDCs vs 1.0 µM TiO_2_** | **-21.12** | **7.676** | **Yes** | ******* | **-34.01 to -8.225** |
| **iDCs vs 10 µM TiO_2_** | **-30.32** | **11.02** | **Yes** | ******* | **-43.21 to -17.43** |
| **iDCs vs 100 µM TiO_2_** | **-37.33** | **13.57** | **Yes** | ******* | **-50.22 to -24.43** |
| **mDCs vs 0.1 µM CeO_2_** | **24.26** | **8.816** | **Yes** | ******* | **11.36 to 37.15** |
| **mDCs vs 1.0 µM CeO_2_** | **24.66** | **8.962** | **Yes** | ******* | **11.76 to 37.55** |
| **mDCs vs 10 µM CeO_2_** | **23.68** | **8.606** | **Yes** | ******* | **10.78 to 36.57** |
| **mDCs vs 100 µM CeO_2_** | **21.32** | **7.748** | **Yes** | ******* | **8.422 to 34.21** |
| **mDCs vs 0.1 µM TiO_2_** | **12.43** | **4.518** | **No** | **ns** | **-0.4633 to 25.32** |
| **mDCs vs 1.0 µM TiO_2_** | **9.005** | **3.273** | **No** | **ns** | **-3.889 to 21.90** |
| **mDCs vs 10 µM TiO_2_** | **-0.1978** | **0.07189** | **No** | **ns** | **-13.09 to 12.70** |
| **mDCs vs 100 µM TiO_2_** | **-7.202** | **2.618** | **No** | **ns** | **-20.10 to 5.692** |
| **0.1 µM CeO_2_ vs 0.1 µM TiO_2_** | **-11.83** | **4.298** | **No** | **ns** | **-24.72 to 1.068** |
| **1.0 µM CeO_2_ vs 1.0 µM TiO_2_** | **-15.65** | **5.689** | **Yes** | ****** | **-28.54 to -2.757** |
| **10 µM CeO_2_ vs 10 µM TiO_2_** | **-23.87** | **8.677** | **Yes** | ******* | **-36.77 to -10.98** |
| **100 µM CeO_2_ vs 100 µM TiO_2_** | **-28.52** | **10.37** | **Yes** | ******* | **-41.41 to -15.62** |
