# Supplementary material for: Immunomodulation and T Helper TH1/TH2 Response Polarization by CeO2 and TiO2 Nanoparticles
Source: PLoS One. 2013 May 8;8(5):e62816. doi: 10.1371/journal.pone.0062816 (PMC3648566; doi:10.1371/journal.pone.0062816)
Supplement: Table S2 — Statistical analysis of Figure 6 B. As in Table S1, Tukey’s honest significance test was employed, in conjunction with an ANOVA, to determine if the treatment groups (between CeO2 and TiO2) are significantly different from each other in relation to CD25 expression. (DOCX) [file pone.0062816.s005.docx]

Supplementary Table S2. Statistical analysis of Figure 6 B.

| **Tukey's Multiple Comparison Test** | **Mean Diff.** | **q** | **Significant? P < 0.05?** | **Summary** | **95% CI of diff** |
| --- | --- | --- | --- | --- | --- |
| **T cells alone vs iDCs** | **-7.948** | **3.206** | **No** | **ns** | **-19.57 to 3.671** |
| **T cells alone vs mDCs** | **-32.84** | **13.25** | **Yes** | ******* | **-44.46 to -21.22** |
| **iDCs vs mDCs** | **-24.89** | **10.04** | **Yes** | ******* | **-36.51 to -13.27** |
| **iDCs vs 0.1 µM CeO_2_** | **-3.506** | **1.414** | **No** | **ns** | **-15.12 to 8.113** |
| **iDCs vs 1.0 µM CeO_2_** | **-7.294** | **2.942** | **No** | **ns** | **-18.91 to 4.325** |
| **iDCs vs 10 µM CeO_2_** | **-6.032** | **2.433** | **No** | **ns** | **-17.65 to 5.587** |
| **iDCs vs 100 µM CeO_2_** | **-8.779** | **3.541** | **No** | **ns** | **-20.40 to 2.840** |
| **iDCs vs 0.1 µM TiO_2_** | **-18.09** | **7.298** | **Yes** | ******* | **-29.71 to -6.473** |
| **iDCs vs 1.0 µM TiO_2_** | **-26.18** | **10.56** | **Yes** | ******* | **-37.79 to -14.56** |
| **iDCs vs 10 µM TiO_2_** | **-30.45** | **12.28** | **Yes** | ******* | **-42.07 to -18.83** |
| **iDCs vs 100 µM TiO_2_** | **-28.84** | **11.63** | **Yes** | ******* | **-40.46 to -17.22** |
| **mDCs vs 0.1 µM CeO_2_** | **21.38** | **8.625** | **Yes** | ******* | **9.765 to 33.00** |
| **mDCs vs 1.0 µM CeO_2_** | **17.6** | **7.097** | **Yes** | ******* | **5.977 to 29.21** |
| **mDCs vs 10 µM CeO_2_** | **18.86** | **7.606** | **Yes** | ******* | **7.239 to 30.48** |
| **mDCs vs 100 µM CeO_2_** | **16.11** | **6.498** | **Yes** | ******* | **4.492 to 27.73** |
| **mDCs vs 0.1 µM TiO_2_** | **6.798** | **2.742** | **No** | **ns** | **-4.821 to 18.42** |
| **mDCs vs 1.0 µM TiO_2_** | **-1.285** | **0.5183** | **No** | **ns** | **-12.90 to 10.33** |
| **mDCs vs 10 µM TiO_2_** | **-5.562** | **2.243** | **No** | **ns** | **-17.18 to 6.057** |
| **mDCs vs 100 µM TiO_2_** | **-3.952** | **1.594** | **No** | **ns** | **-15.57 to 7.667** |
| **0.1 µM CeO_2_ vs 0.1 µM TiO_2_** | **-14.59** | **5.883** | **Yes** | ****** | **-26.20 to -2.967** |
| **1.0 µM CeO_2_ vs 1.0 µM TiO_2_** | **-18.88** | **7.616** | **Yes** | ******* | **-30.50 to -7.262** |
| **10 µM CeO_2_ vs 10 µM TiO_2_** | **-24.42** | **9.85** | **Yes** | ******* | **-36.04 to -12.80** |
| **100 µM CeO_2_ vs 100 µM TiO_2_** | **-20.06** | **8.093** | **Yes** | ******* | **-31.68 to -8.444** |
